# A New Mathematical Model to Index Body Weight in Healthy Chinese Han Adults

**DOI:** 10.1002/mco2.70649

**Published:** 2026-03-02

**Authors:** Qing Zhang, Gui‐Hua Yao, Xiang‐Yun Chen, Mei Zhang, Xueying Zeng, Shuping Wang, Cheng Zhang, Yun Zhang

**Affiliations:** ^1^ State Key Laboratory For Innovation and Transformation of Luobing Theory The Key Laboratory of Cardiovascular Remodeling and Function Research Chinese Ministry of Education Chinese National Health Commission Chinese Academy of Medical Sciences and Shandong Province Department of Cardiology Qilu Hospital of Shandong University Jinan China; ^2^ Department of Cardiology Qilu Hospital (Qingdao) Cheeloo College of Medicine Shandong University Qingdao China; ^3^ Department of Cardiology Qilu Hospital School of Medicine Cheeloo College of Medicine Shandong University Jinan Shandong China; ^4^ School of Mathematical Sciences Ocean University of China Qingdao China; ^5^ Laboratory of Marine Mathematics Ocean University of China Qingdao China; ^6^ Department of Endocrinology and Metabolism Dongying People's Hospital Dongying China

**Keywords:** body mass index, diagnostic criterion of overweight, optimized multivariate allometric model, overweight

## Abstract

Body mass index (BMI) is traditionally used to diagnose overweight and obesity, but it is influenced by physiological variables. This study tested the hypothesis that body weight is nonlinearly related to age and height, and that an optimized multivariate allometric model (OMAM) could correct for these effects and define a new criterion for overweight diagnosis. A total of 1498 Chinese Han adults were enrolled. The normal weight group (BMI < 25.0 kg/m^2^, *n* = 1224) was divided into subgroup A (*n* = 857) to develop OMAM equations and determine the threshold, and subgroup B (*n* = 367) to validate them. The overweight group (BMI ≥ 25.0 kg/m^2^, *n* = 274) was used to test the new criterion. OMAM corrected the nonlinear influence of age, height, and sex on weight. A corrected weight value $W_{C}$>1.1440 was defined as the new threshold. This criterion reclassified 21.9% of overweight individuals as normal weight and reduced false positives, notably lowering the overweight rate to 61.3% in men, while minimizing unnecessary interventions. Compared with BMI, the new criterion showed higher specificity and accuracy in identifying diabetes, hypertension, coronary heart disease, and metabolic syndrome in the external CAPITAL cohort. These findings support the clinical utility of OMAM in overweight screening. Further validation in non‐Chinese Han populations is warranted.

## Graphical abstract

We developed an optimized multivariate allometric model to correct the effects of age, sex, and height on body weight. A new index *W*_c_ > 1.1440 was established as a criterion for diagnosing overweight in Chinese Han adults. This criterion reduces overweight misclassification in men and provides a more specific assessment of cardiometabolic risk.

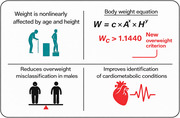

## 1 Introduction

Over the last 30 years, the prevalence of overweight and obesity has doubled worldwide, leading to a surge in metabolic diseases, such as metabolic syndrome, diabetes, hypertension, and atherosclerosis, and posing a serious threat to public health [1]. Thus, early and precise diagnosis of overweight and obesity is of great importance for the prevention and treatment of metabolic disease in adult populations.

In 1832, Adolphe Quetelet, a Belgian mathematician, measured body weight in an Anglo‐Saxon population and found that weight was related to the square of height, and an individual's weight divided by the square of his/her height was termed as Quetelet index [2]. In 1972, Ancel Keys, an American physiologist, examined several height–weight indices and found that the Quetelet index was the best predictor of the thickness of body fat, which was renamed as body mass index (BMI) [3]. Since then, BMI has gained wide clinical applications and recommendations by many guidelines as diagnostic criterion of overweight and obesity, preventive and therapeutic target for obesity, atherosclerotic cardiovascular disease (ASCVD) [4, 5, 6, 7, 8, 9], and prognostic indicator of diabetes and ASCVD [10, 11, 12, 13, 14, 15, 16, 17]. A recent study reported that a high BMI (BMI > 22.5 kg/m^2^) led to multiple diseases including cardiovascular disease, diabetes, and cancer [18, 19]. Based on these studies, the World Health Organization (WHO) recommended BMI ≥ 25.0 kg/m^2^ as a criterion for diagnosing overweight [2, 20].

In contrast to these paeans to BMI, recent studies began to question the reliability of BMI as an indicator of health. The accuracy of BMI in reflecting body fat content or cardiometabolic risk has been increasingly challenged [21, 22, 23]. Using body fat percent measurement as a standard, BMI had a poor performance in the diagnosis of obesity [24, 25, 26, 27, 28]. In the National Health and Nutrition Examination Survey, about 30% of obese subjects were cardiometabolically healthy (determined by blood pressure and serum cholesterol), while the same percentage of subjects with a healthy BMI were considered cardiometabolically unhealthy [29]. Das et al. investigated 50,000 patients with ST‐segment elevation myocardial infarction and found that the adjusted odds of death were lowest for Class I obese patients (30.0 kg/m^2 ^≤ BMI < 35.0 kg/m^2^), whereas no significant difference in mortality was observed between the normal‐weight and overweight (25.0 kg/m^2 ^≤ BMI < 30.0 kg/m^2^) [30]. This finding demonstrated the inability of traditional BMI to differentiate the risk of adverse outcomes between normal‐weight and overweight individuals. A study of over 1 million Asians demonstrated that the risk of death was lowest within a broad BMI range of 22.6–27.5 kg/m^2^, further supporting that risk did not significantly differ between the healthy and overweight categories [31]. In addition, more recent studies found that the risk of death in overweight adults was similar to that in healthy adults, lending support to previous findings [32, 33, 34]. These discrepancies underscore an urgent need for a more precise diagnostic standard to accurately define overweight and better predict individual cardiometabolic risks.

The reason for the discrepancies in the clinical values of BMI among different studies is unclear but one possible explanation is that traditional BMI formula did not take into account the effect of important physiological factors such as sex, age, or ethnicity on body weight, leading to a wide dispersion of normal values and erroneous definition of cutoffs [23, 35]. Several studies have provided supportive evidence for this explanation by demonstrating the gender bias in BMI‐based classification, with men being more likely to be overdiagnosed due to higher values than women in height, fat distribution, and muscle mass [36, 37, 38].

In the Echocardiographic Measurements in Normal Chinese Adults (EMINCA) study, we recruited 1394 healthy Han Chinese volunteers aged 18–79 years throughout China using strict inclusion and exclusion criteria and developed an optimized multivariate allometric model (OMAM) for correcting two‐dimensional and Doppler echocardiographic measurements for physiological variables [39, 40, 41, 42]. The results of the EMINCA study showed that the weight of healthy Chinese Han adults differed between genders and correlated with age and height, whereas the BMI formula considered only the effect of height on weight. This highlighted the need for a weight index that could eliminate physiological confounders and better reflect individualized health risk. Thus, the purpose of the present study is fourfold: first, to develop an OMAM equation to correct the effect of age, gender, and height on weight in the EMINCA population; second, to establish a new criterion for the diagnosis of overweight based on the OMAM equation; third, to examine the difference in the incidence of overweight detected by traditional BMI and by the new criterion in a separate group of population, and finally, to compare the performance between the new and old diagnostic criteria of overweight in identifying cardiometabolic conditions including diabetes mellitus, hypertension, coronary heart disease, and metabolic syndrome in an independent Carotid Artery Plaque Intervention with Tongxinluo Capsule (CAPITAL) study cohort.

## 2 Results

A total of 1498 healthy Chinese Han subjects participated in the study, including 1224 normal weight subjects and 274 overweight subjects. The demographic characteristics of the normal weight group were shown in Table 1. There was no difference in age between men and women. However, height, weight, and BMI were all significantly higher in men than in women. It is important to note that BMI did not correct the effect of gender on weight. The demographic characteristics of the overweight group were also shown in Table 1. There was no difference in age and BMI between men and women. However, height and weight were also significantly higher in men than in women.

**TABLE 1:** Demographic characteristics of men and women in the normal weight and overweight groups.

	Men (*n* = 579)	Women (*n* = 645)	*p* Value
Normal weight			
Age	46 (28)	46 (26)	0.676
Height	1.71 (0.07)	1.60 (0.07)	<0.001**
Weight	65.90 (9.40)	55.10 (9.00)	<0.001**
BMI	22.65 (2.76)	21.48 (3.10)	<0.001**

	Men (*n* = 137)	Women (*n* = 137)	*p* Value
Overweight			
Age	50 (22)	50 (22)	0.306
Height	1.72 (0.07)	1.61 (0.07)	<0.001**
Weight	77.00 (7.90)	68.10 (6.55)	<0.001**
BMI	25.93 (1.98)	25.91 (1.36)	0.146

Data were median (IQR) or *p* value. **p<0.01.

*Abbreviation*: BMI: body mass index.

In the normal weight group, if the indexation with BMI is successful, after dividing body weight by squared height, BMI should no longer correlate with any physiological variables that affect weight. However, as shown in Table 2, BMI still correlated significantly with height and age in 1224 normal weight subjects, although difference existed in these correlations between sexes. Hence, indexation with BMI did not eliminate the effect of height and age on body weight.

**TABLE 2:** Correlation of BMI with weight, height, and age in the normal weight group.

	Weight	Height	Age
Population (*n* = 1224)	0.724, <0.001**	0.173, <0.001**	0.166, <0.001**
Men (*n* = 579)	0.760, <0.001**	0.099, 0.017*	0.077, 0.066
Women (*n* = 645)	0.781, <0.001**	−0.107, 0.007**	0.261, <0.001**

Data were ρ, *p* value. **p<0.01.

*Abbreviation*: BMI: body mass index.

The demographic characteristics of subgroup A and subgroup B were shown in Table 3. The proportion of men in subgroup A and subgroup B was 47.3 and 47.4%, respectively, presenting no significant difference in sex ratio between the two subgroups. In addition, there was no significant difference in age, height, weight, and BMI between the two subgroups, indicating a success in randomization.

**TABLE 3:** Demographic characteristics of subjects in subgroup A and subgroup B.

	Subgroup A (*n* = 857)	Subgroup B (*n* = 367)	*p* Value
Age	46 (27)	47 (27)	0.729
Height	1.65 (0.11)	1.65 (0.11)	0.979
Weight	60.00 (12.00)	60.00 (12.00)	0.968
BMI	22.10 (3.07)	22.09 (3.11)	0.855

Data were median (IQR) or *p* value.

*Abbreviation*: BMI: body mass index.

### 2.1 Development of OMAM Equations in Normal Weight Group and the New Criterion for Overweight Diagnosis

We developed the OMAM equations for indexing of body weight and established a new criterion for diagnosing overweight based on the study of the normal weight group. The OMAM equations developed in subgroup A were shown as Equations (1) and (2). The corrected value in subgroup A was the measured value divided by the predicted value $W_{P}$ derived from the OMAM equations. The median (IQR) of the corrected value $W_{C}$ in subgroup A was 1.0036 (0.1253). The upper limit of the normal reference range, defined as the 97.5th percentile, was 1.1440. As a result, we suggest that $\;W_{C}\;$> 1.1440 should be the new criterion for diagnosing overweight in Chinese Han adults. Specifically, an individual is classified as having normal weight if his/her $W_{C\;}$≤ 1.1440. Otherwise, he/she will be deemed as overweight.

### 2.2 Verification of OMAM Equations and New Criterion in Subgroup B

We applied the OMAM equations developed in subgroup A to subgroup B and calculated the predicted value $W_{P}$ and the corrected value $W_{C}$ in the latter group. As shown in Table 4, the median (IQR) of the corrected value $W_{C}$ in subgroup B was 1.0020 (0.1314), which was similar to that in subgroup A. In addition, $W_{C}$ was correlated with the actually measured weight (ρ= 0.557, p< 0.001) in subgroup B. Moreover, $W_{C}$ was uncorrelated with age and height (p > 0.05). Finally, the median (IQR) of $W_{C}$ was 1.0046 (0.1247) for men and 0.9996 (0.1374) for women in subgroup B, and Mann–Whitney *U* test showed no significant difference between men and women (p= 0.712). Thus, the OMAM equations developed in subgroup A can be extrapolated to subgroup B for weight prediction, as they satisfied the three conditions predefined in mathematical analysis. We then examined subjects in subgroup B using $W_{C\;}$> 1.1440 as the new criterion for overweight diagnosis and found that only nine of 367 subjects (2.45%) had $W_{C\;}$> 1.1440. A binomial test indicated no significant difference between the observed proportion 2.45% and the theoretical proportion 2.5% (p= 0.941), confirming that the threshold effectively met the requirement for 95% confidence.

**TABLE 4:** Validation of OMAM equations in subgroup B.

	Correlation between $W_{C}$ and weight, age, or height		
$W_{C}$	Weight	Age	Height
1.0020 (0.1314)	0.557, <0.001**	−0.043, 0.417	−0.023, 0.656

Data were median (IQR) or ρ, *p* value. **p<0.01.

*Abbreviation*: $W_{C}$: corrected value of weight.

### 2.3 Application of OMAM Equations and New Criterion to Overweight Group

**FIGURE 1:**
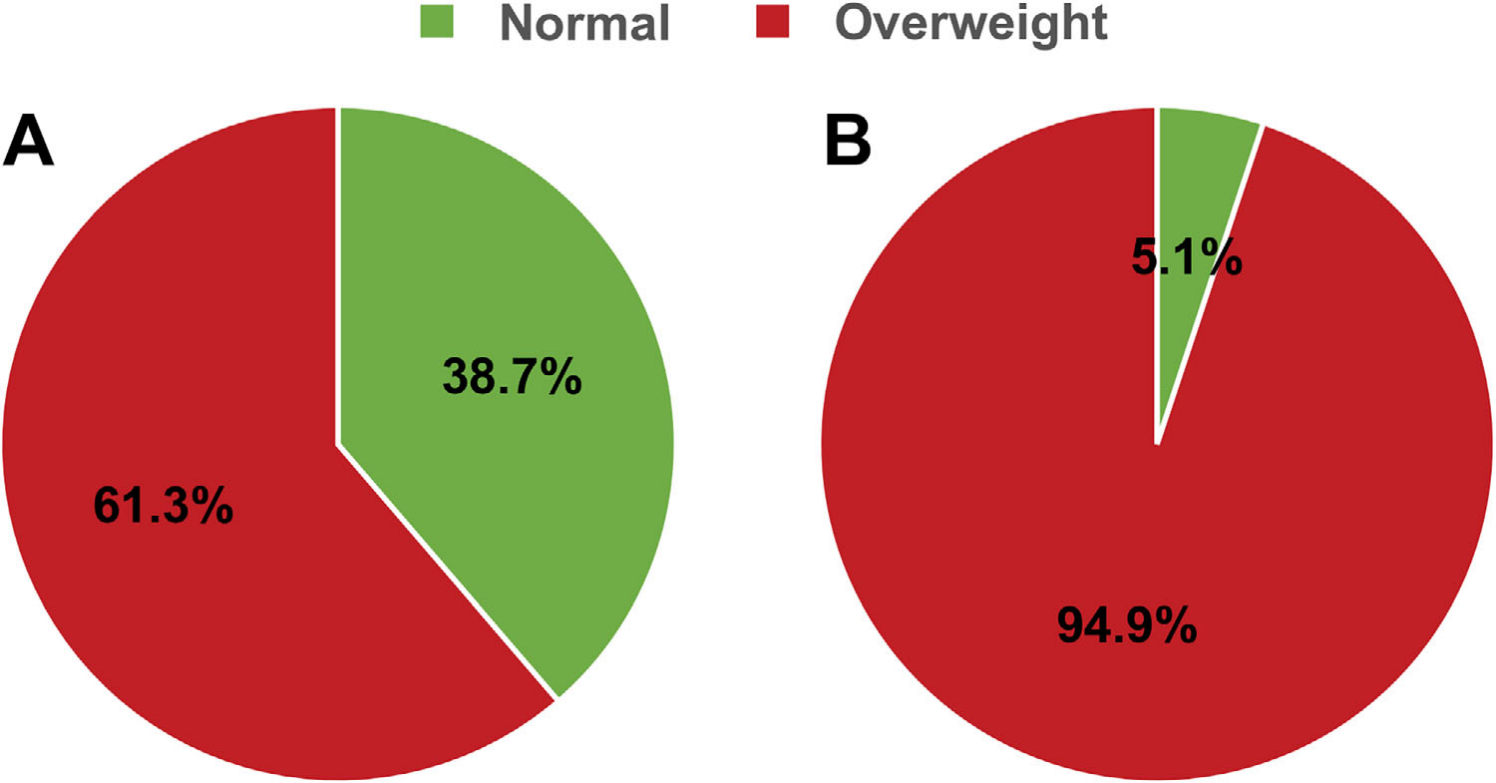
Application of the new criterion for diagnosing overweight in the overweight group. (A) Among the 137 men with BMI ≥ 25 kg/m^2^, 38.7% (*n* = 53) were reclassified as normal weight subjects using the new criterion. (B) Among the 137 women with BMI ≥ 25 kg/m^2^, 5.1% (*n* = 7) were reclassified as normal weight subjects using the new criterion.

To assess the performance of the OMAM equations and the new overweight diagnosis criterion, we applied them to the subjects in the overweight group (BMI ≥ 25.0 kg/m^2^, *n* = 274, including 137 men and 137 women). After calculation, the median (IQR) of the corrected value $W_{C}$ was 1.1835 (0.0756) when the weight of the overweight group was predicted using the OMAM equations. Wilcoxon signed‐rank test indicated that the predicted value $W_{P}$ was significantly lower than the measured value (*p* < 0.001). In addition, the median (IQR) of $W_{C}$ was 1.1550 (0.0805) for men and 1.1986 (0.0615) for women, with a significant difference in the corrected value of $W_{C}$ between genders (*p *< 0.001). The median of the corrected value $W_{C}\;$ for men was obviously lower than for women. Then, we applied the new criterion for diagnosis of overweight ($W_{C}\;$> 1.1440) to the overweight group. The results showed that 60 subjects had a corrected value of $W_{C}$ not larger than 1.1440 among the 274 subjects. Consequently, 21.9% of overweight subjects defined by BMI ≥ 25.0 kg/m^2^ were reclassified as having normal weight, and the overweight rate was reduced to 78.1% when using our new criterion. Of these 60 misclassified subjects, 53 were men and seven were women, indicating a trend for overdiagnosis of overweight in men when using BMI criteria. Therefore, compared with the traditional criterion (BMI ≥ 25.0 kg/m^2^), the new criterion reduced the overweight rate to 61.3% in men and to 94.9% in women in the overweight group (Figure 1).

### 2.4 Application of New Criterion to CAPITAL Study Group

To further evaluate the clinical value of the new criterion in detecting overweight ($W_{C\;}$> 1.1440), we applied both the new criterion and BMI to an independent external cohort in the CAPITAL study. This cohort consisted of 1219 Chinese Han adults (727 men and 492 women) with complete demographic characteristics, biochemical measurements, and medical history, in whom 241, 659, 272, and 262 subjects had diabetes mellitus, hypertension, coronary heart disease, and metabolic syndrome, respectively. The performance of the new criterion and BMI for detecting these four conditions was compared based on analysis of sensitivity, specificity, and accuracy.

**FIGURE 2:**
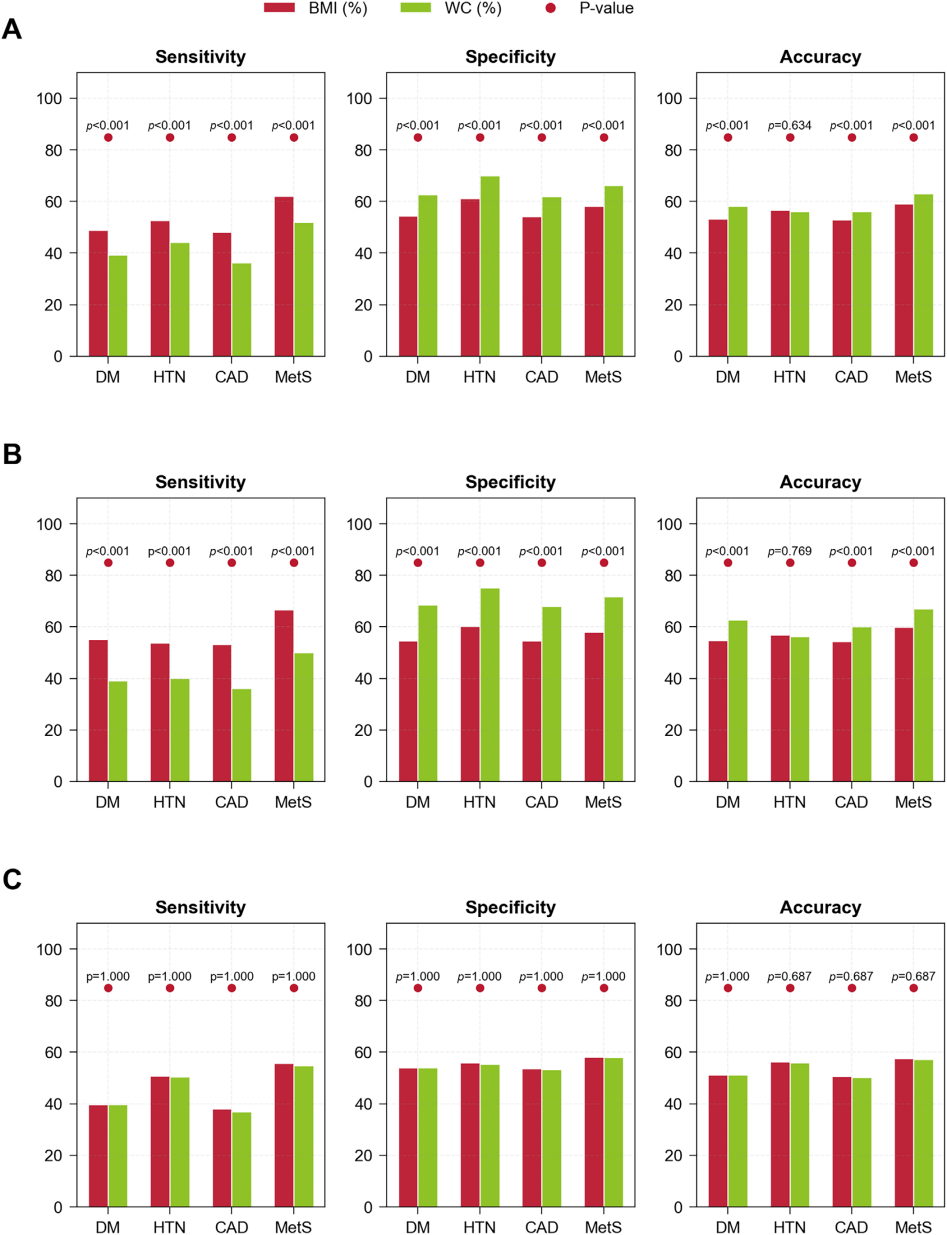
Sensitivity, specificity and accuracy of $W_{C}$‐based and BMI‐based criteria in CAPITAL study cohort for diagnosing diabetes mellitus (DM), hypertension (HTN), coronary artery disease (CAD), and metabolic syndrome (MetS). (A) Entire cohort. (B) Males. (C) Females.

As shown in Table 5 and Figure 2, in the total cohort and among male participants in the CAPITAL study, the $W_{C}$‐based criterion demonstrated consistently higher specificity across all four cardiometabolic diseases compared with the traditional BMI criterion (*p *< 0.05). This highlighted the ability of $W_{C}$ to reduce false‐positive classification of overweight individuals. In terms of accuracy, the $W_{C}$‐based criterion also outperformed BMI‐based criterion in identifying diabetes mellitus, coronary heart disease, and metabolic syndrome (*p *< 0.05). However, the BMI‐based criterion showed higher sensitivity across all four diseases, suggesting that it may capture more true‐positive cases but at the cost of more false positives. Notably, among female participants, the diagnostic performance of the $W_{C}$‐based and BMI‐based criteria was almost identical across all metrics and disease categories, indicating gender‐related differences in the effectiveness of these two overweight criteria in identifying cardiometabolic diseases. Nonetheless, as men are more vulnerable to cardiometabolic diseases than women, the high accuracy of *W*_C_‐based criteria in men is of paramount clinical importance.

**TABLE 5:** Application of new criterion and BMI in CAPITAL cohort.

	Metric	DM			HTN			CAD			MetS		
		BMI	$W_{C}$	*p* Value	BMI	$W_{C}$	*p* Value	BMI	$W_{C}$	*p* Value	BMI	$W_{C}$	*p* Value
Total (*n* = 1219)	Accuracy	53.32%	58.08%	<0.001**	56.60%	56.11%	0.634	52.88%	56.17%	<0.001**	59.03%	63.14%	<0.001**
	Sensitivity	48.96%	39.42%	<0.001**	52.66%	44.31%	<0.001**	48.16%	36.40%	<0.001**	62.21%	51.91%	<0.001**
	Specificity	54.4%	62.68%	<0.001**	61.25%	70.00%	<0.001**	54.24%	61.86%	<0.001**	58.16%	66.21%	<0.001**
Male (*n* = 727)	Accuracy	54.75%	62.72%	<0.001**	56.81%	56.26%	0.769	54.34%	60.14%	<0.001**	59.92%	67.08%	<0.001**
	Sensitivity	55.24%	39.16%	<0.001**	53.83%	40.05%	<0.001**	53.33%	36.11%	<0.001**	66.67%	50.00%	<0.001**
	Specificity	54.62%	68.49%	<0.001**	60.30%	75.22%	<0.001**	54.68%	68.07%	<0.001**	58.07%	71.75%	<0.001**
Female (*n* = 492)	Accuracy	51.22%	51.22%	1.000	56.30%	55.89%	0.687	50.71%	50.31%	0.687	57.72%	57.32%	0.687
	Sensitivity	39.80%	39.80%	1.000	50.94%	50.56%	1.000	38.04%	36.96%	1.000	55.66%	54.72%	1.000
	Specificity	54.06%	54.06%	1.000	55.85%	55.44%	1.000	53.63%	53.38%	1.000	58.29%	58.03%	1.000

**p<0.01.

*Abbreviations*: BMI, body mass index; *W*_c_, corrected value of weight; DM, diabetes mellitus; HTN, hypertension; CAD, coronary artery disease; MetS, metabolic syndrome.

## 3 Discussion

There were several important findings in the present study. First, among Chinese Han healthy adults, body weight exhibited a significant difference between men and women and correlated nonlinearly with age and height. Second, indexation with BMI did not eliminate the effect of age, gender, and height on body weight. Third, OMAM equations successfully corrected the effects of age, gender, and height on body weight. Fourth, $W_{C}$ > 1.1440 was established as a new criterion for the diagnosis of overweight. Fifth, relative to the traditional criterion (BMI ≥ 25.0 kg/m^2^), the new criterion reduced the overweight rate from 100 to 61.3% for men and from 100 to 94.9% for women in a Chinese overweight population. Finally, when applied to an external CAPITAL study cohort, $W_{C}$‐based criterion outperformed BMI in terms of specificity and overall accuracy for identifying individuals with diabetes, hypertension, coronary heart disease, and metabolic syndrome. These findings suggest that the $W_{C}$‐based criterion offers a more precise tool for overweight classification and early cardiometabolic risk screening in the Chinese population.

Overweight and obesity, as a chronic metabolic syndrome, are recognized risk factors of several global epidemics including hypertension, diabetes, and dyslipidemia [43]. Since Quetelet first proposed BMI in the 19th century [44], it has been widely used to evaluate the severity of adiposity, but recent studies cast doubt on BMI as an indicator of risk and prognosis of ASCVD [24, 25, 45]. As a result, enthusiasm for BMI has begun to wane [34]. In 2023, the American Medical Association, recognizing BMI as an “imperfect measure,” called for the downplaying of the clinical use of BMI [23].

BMI, defined as an individual's weight in kilograms (kg) divided by the square of height in meters (m^2^), only accounts for the effect of height on weight, but our study showed that age, sex, and height all had a significant effect on weight. The effects of age and height on weight are also physiologically justified, as aging is accompanied by changes in body composition such as fat redistribution and muscle loss, while height reflects skeletal and muscular frame size, both of which influence body weight. Previous studies have shown that the risk of ischemic heart disease resulting from obesity was different between sexes [46]. In addition, the prevalence of overweight and obesity was significantly higher and the BMI values grew faster in men than in women, indicating a gender difference in BMI trends [47]. In addition to physiological variables, ethnicity also exerted an important effect on weight due to genetic and environmental factors [48]. Bajaj et al. reported that due to a higher prevalence of central obesity, Asian people with a lower BMI may face a higher risk of type 2 diabetes and ASCVD than other races, and thus a race‐specific adjustment to the BMI threshold of obesity has been proposed for Asians [49]. Because a number of physiological variables may affect the value of weight, simple indexation with BMI is inadequate. Table 2 shows that BMI still correlated with height and age, demonstrating that BMI is not an ideal indexing tool, and the diagnostic criteria of overweight and obesity based on BMI are not valid. Therefore, a new criterion for the diagnosis of overweight that is independent of gender, age, and height is highly warranted for the Chinese people.

In this study, we hypothesized that there exists a nonlinear relationship between an individual's weight and his/her height and age, and introduced gender into the regression equation in the form of a dummy variable to develop a multiple regression model. The significance tests of the model and coefficients were successfully passed with a goodness‐of‐fit adjusted $\;R^{2}$ of 0.6634, representing good fitting results and explanatory power. In addition, we verified that the corrected value $W_{C}$, which was calculated by dividing the measured value W by the predicted value $W_{P}$, completely removed the effects of gender, age, and height on weight (*p *> 0.05).

### 3.1 Development of the New Criterion for Diagnosis of Overweight

The international cut‐off value between normal weight and overweight was initially determined based on studies of total mortality in western populations, and BMI values with lower mortality was regarded as a normal range [20]. Later on, the incidence of obesity‐associated diseases was used to determine the cut‐offs of overweight so as to prevent these chronic diseases as early as possible [50]. Recently, WHO has divided BMI into three categories: BMI 18.50–24.99 kg/m^2^ as normal weight, BMI 25.0–29.9 kg/m^2^ as overweight, and BMI ≥ 30.0 kg/m^2^ as obesity. However, the definition of the three categories of BMI was derived mainly from studies of western populations [20]. Considering the effect of races on body weight, the International Association for the Study of Obesity, the International Obesity Task Force and WHO defined BMI of 23.0–24.9 kg/m^2^ as overweight and BMI ≥ 25.0 kg/m^2^ as obesity in Asian adults [51]. In a cross‐sectional sample of 15,239 Chinese adults, by taking into account of the prevalence of hypertension, diabetes, and dyslipidemia, Wildman et al. recommended BMI of 24.0 kg/m^2^ as an optimal cut‐off point for diagnosis of overweight [4]. These studies demonstrated the inconsistency of the cut‐off value of BMI for the diagnosis of overweight and obesity in the Chinese population. Therefore, it is of great importance to determine the normal values of body weight in healthy Chinese adults, and on this basis, define the optimal cut‐off value for identifying overweight and obesity in the Chinese population.

To overcome the limitations of traditional BMI, we developed the OMAM equations in this study, which successfully removed the effects of gender, age, and height on weight. The median (IQR) of the corrected value $W_{C}$ in our normal weight group were calculated as 1.0036 (0.1253) using the OMAM equations. The 97.5th percentile of $W_{C}$ was 1.1440, which was therefore established as the new criterion for diagnosing overweight in Chinese Han adults.

### 3.2 Application of the New Criterion in the Overweight Group

We applied the new diagnostic criterion ($W_{C}$>1.1440) to an overweight population of 274 individuals (137 men and 137 women) with a BMI ≥ 25.0 kg/m^2^. Consequently, 60 individuals (21.9%) were reclassified as normal weight by the new criterion. Specifically, as shown in Figure 1, 53 men and seven women had $W_{C\;}$≤ 1.1440, indicating that the overweight rate decreased from 100 to 61.3% in men and from 100 to 94.9% in women. These findings demonstrate that the $W_{C}$‐based criterion is more conservative and specific than the BMI‐based criterion in identifying truly overweight individuals.

Despite the balanced sex distribution and the absence of significant differences in BMI between men and women in the overweight group (Table 1), a higher proportion of men than women were reclassified as having normal weight under the new criterion. This gender discrepancy may be partially explained by two inherent limitations of the traditional BMI system. First, the BMI formula was originally developed based on Western populations and assumed a quadratic relationship between weight and height (i.e., weight divided by height squared). However, in our OMAM Equations (1) and (2), derived from a large Chinese Han cohort, the optimal exponent for height was found to be 2.1659, which is higher than 2. This suggests that a stronger height adjustment is required for this population. As a result, the BMI formula may underestimate the influence of height in taller individuals, most of whom are men, and thus systematically penalizes them with falsely elevated BMI values. Second, BMI neglects the physiological differences in age and sex [23, 35]. Notably, BMI overlooks sex‐specific variations in body composition [36]. At the same BMI, men generally have more lean mass and less subcutaneous fat than women, leading to overestimation of adiposity in men [37, 38]. Recent studies have also reported similar findings, showing that BMI tends to overestimate body fat in men and underestimate it in women, resulting in gender disparities in overweight diagnosis [36].

From a clinical and public health perspective, this gender bias has meaningful implications. Overdiagnosis of overweight in metabolically healthy men may lead to unnecessary interventions and increased healthcare costs [22]. In contrast, the OMAM‐derived metric corrects for the effects of age, sex, and height, offering a more individualized and physiologically grounded assessment. By accounting for ethnic, sex‐specific, and age‐related variations in body size and composition, the OMAM metric addresses the inherent limitations of traditional BMI and holds promise for improving diagnostic precision and reducing misclassification in both clinical screening and epidemiological surveillance.

However, our results also revealed a significant gender disparity in $W_{C}$ within the overweight group, with women exhibiting higher values than men. This discrepancy suggests that the OMAM equations, originally developed using a normal weight cohort, may systematically underpredict weight in overweight women to a greater extent than in men. This sex‐biased performance indicates that the physiological relationship between predicted and measured weight may shift differently across genders when transitioning to a pathological state (overweight).

### 3.3 Application of New Criterion in CAPITAL Group

The clinical utility of the $W_{C}$‐based criterion ($W_{C}\;$> 1.1440) was further evaluated in an independent CAPITAL study cohort, where its diagnostic performance was compared with that of the traditional BMI‐based criterion (BMI ≥ 25.0 kg/m^2^) for identifying four major cardiometabolic diseases: diabetes mellitus, hypertension, coronary heart disease, and metabolic syndrome.

The $W_{C}$ criterion demonstrated significantly higher specificity and overall accuracy than BMI, particularly in the total population and male subgroup. This finding indicated that $W_{C}$ can more effectively reduce false‐positive overweight diagnoses, which is crucial in large‐scale screening and preventive healthcare settings where minimizing unnecessary interventions is essential. These results lent support to prior critiques of BMI's limitations in distinguishing between fat distribution and metabolic status across sexes and ages [23, 36].

In contrast, the BMI‐based classification consistently showed higher sensitivity, suggesting it may capture more true‐positive cases, but at the cost of a higher false‐positive rate. This implies that BMI may still be suitable in certain clinical contexts, such as follow‐up of high‐risk individuals, where failing to identify any potentially at‐risk subjects is more detrimental than overidentifying them. This diagnostic trade‐off between sensitivity and specificity underscores the complementary nature of these two criteria [52].

Interestingly, in the female subgroup, both $W_{C}$ and BMI performed similarly across all metrics, echoing evidence that body composition, fat distribution, and height variability were less pronounced among women than among men, thereby resulting in fewer classification discrepancies [37].

Taken together, these results supported $W_{C}$ as a more precise and practical weight‐based screening tool, particularly in the general population and among male individuals, where BMI tends to overestimate overweight prevalence. The application of $W_{C}$ may improve the cost effectiveness of public health screening programs, reduce psychological and economic burdens on metabolically healthy individuals, and enable more targeted downstream clinical evaluations. Future guidelines may consider incorporating both indices based on clinical context, using $W_{C}$ for population‐level assessments and BMI for clinical follow‐up, to optimize their respective strengths in stratifying disease risk.

## 4 Study Limitations

The present study contains several limitations. First, the study population consisted of only Chinese Han adults because Han accounts for 92% of the entire Chinese, and it is unclear whether this method can correct the effect of age, gender, and height in other races or eliminate the effect of race on weight, which requires further investigation. Second, the OMAM equations were developed using a metabolically healthy population. Whether these equations can be directly applied to individuals with overweight, obesity, or comorbidities such as diabetes or cardiovascular disease requires further investigation. Third, although we performed external validation in an independent CAPITAL cohort, the long‐term predictive value of the new overweight criterion for adverse health outcomes (e.g., mortality, cardiovascular events) was not assessed. Future prospective cohort studies are therefore needed to address this gap. Fourth, while the OMAM‐based $W_{C}$ metric demonstrated improved specificity and overall accuracy in cardiometabolic disease screening compared with BMI, its relatively lower sensitivity may restrict its utility in high‐risk clinical follow‐up settings. Fifth, we did not directly compare the $W_{C}$‐based classification with alternative anthropometric indicators such as body fat percentage or waist‐to‐height ratio, mainly because the role of these relative new indices in the prevention of cardiovascular disease has not been established. Sixth, although deriving corrected weight values $W_{C}$ using the OMAM equations may seem time consuming in practice, this limitation has been addressed by providing a user‐friendly online calculator (available at https://xueyingzeng.github.io/OMAMcal/) and a detailed supplementary instruction document. These tools significantly improve the method's accessibility and clinical scalability. We believe that once these limitations are overcome, the new $W_{C}$ criterion will be integrated into existing clinical guidelines and preventive strategy. Finally, a major limitation of this study was the potential gender bias in the model's generalizability. The higher $W_{C}$ in overweight women suggests that the current OMAM model may not fully account for gender‐specific fat distribution patterns in overweight populations. Future studies are required to develop population‐specific calibrations to ensure the model's accuracy across diverse pathological states.

## 5 Conclusion

In healthy Chinese Han adults, body weight showed significant differences between sexes and correlated nonlinearly with age and height. Indexation with BMI did not eliminate these effects, whereas the OMAM equations successfully corrected for the influences of age, gender, and height on body weight. A corrected weight value of $W_{C}\;$> 1.1440 was established as a new criterion for overweight diagnosis, which reduced the false positives of overweight in the EMINCA population. Validation in the external CAPITAL cohort demonstrated that this new criterion outperformed BMI in terms of specificity and overall accuracy in detecting cardiometabolic disease, particularly in male participants, highlighting the clinical value of the $W_{C}$‐based approach in reducing false positives and improving screening precision for population‐level prevention strategies.

## 6 Materials and Methods

### 6.1 Study Population

The study population consisted of three groups. The first group involved the EMINCA population [39, 41], which comprised a total of 1224 healthy Chinese Han subjects (579 men and 546 women) with BMI < 25.0 kg/m^2^ (normal weight group). All participants were recruited from tertiary hospitals in all provinces and municipalities in the mainland of China. The inclusion criteria required volunteers to be aged 18–79 years, have Han nationality, have normal blood pressure (systolic blood pressure < 140 mmHg and diastolic blood pressure < 90 mmHg), have normal results on physical examination, electrocardiography, and echocardiography, and have no history of cardiovascular diseases. The exclusion criteria were coronary artery disease, structural heart disease, heart failure, hypertension, stroke, hyperlipidemia (serum total cholesterol ≥ 5.72 mmol/L or triglyceride ≥ 1.70 mmol/L), diabetes mellitus (fasting blood glucose > 7.0 mmol/L), obesity and any other endocrine diseases, acute or chronic respiratory diseases, anemia, connective tissue disease, abnormal liver function, abnormal renal function, abnormal results on electrocardiography, valvular stenosis, more than mild valvular regurgitation, or wall motion abnormalities on echocardiographic recordings. Professional athletes, pregnant or lactating women, subjects with alcohol abuse, and subjects with inadequate echocardiographic images were also excluded [53]. The study protocol was approved by the ethical committees of all collaborating hospitals, and a written informed consent was obtained from all volunteers participating in this study.

In the EMINCA study, height and weight were measured in all volunteers by clinicians. BMI was calculated as body weight in kg divided by the square of height in meters. To develop a reliable OMAM equation and establish a new criterion for diagnosing overweight, we randomly allocated the 1224 healthy volunteers into two subgroups with a ratio of 7:3 to ensure no significant difference in gender, age, height, and weight between the two groups. Specifically, subgroup A (70%, *n* = 857, 405 men and 452 women) was used to develop an OMAM equation and a new criterion for the diagnosis of overweight, and subgroup B (30%, *n* = 367, 174 men and 193 women) was used to verify the reliability of the new equation and criterion.

The second group of the study population involved 274 overweight subjects, defined by BMI ≥ 25.0 kg/m^2^, including 137 men and 137 women aged 19–79 years (overweight group). All subjects in this group met inclusion and did not meet the exclusion criteria of the EMINCA study except for overweight.

The third group of the study population was derived from the CAPITAL study [54], which was a large‐scale, multicenter, randomized, double‐blind, placebo‐controlled trial conducted in 35 hospitals across 18 provinces in China. The inclusion criteria included men or women aged 40–75 years with noncalcified plaque defined as a focal thickening of the carotid intima‐media thickness of 1.2–3.5 mm in bilateral common or internal carotid arteries detected by ultrasonography. The major exclusion criteria were a history of myocardial infarction and stroke within the past 6 months, a history of percutaneous coronary intervention or carotid endarterectomy, uncontrolled diabetes mellitus or hypertension, familial hypercholesterolemia, Takayasu arteritis, systemic disease, liver or renal dysfunction, pregnancy or lactation, and a requirement for warfarin for anticoagulant therapy or a history of receiving any other investigational traditional Chinese medicine within 12 months before the baseline visit. The study enrolled a total of 1219 participants aged 40–75 years with a median age of 61 years, in whom baseline data including demographic characteristics, biochemical measurements, and medical history were collected, and ultrasonographic assessment of bilateral carotid arteries was performed. There were 241 subjects with diabetes (male 143), 272 subjects with coronary artery disease (male 180), 659 subjects with hypertension (male 392), and 262 subjects with metabolic syndrome (male 156). The baseline data of these patients were used to evaluate the discriminatory performance of the new overweight criterion in comparison with BMI ≥ 25.0 kg/m^2^ for identifying individuals with diabetes mellitus, hypertension, coronary heart disease, and metabolic syndrome.

### 6.2 Mathematical Analysis

After plotting the curves of weight with age or height in the EMINCA data, we found that there existed a nonlinear relationship between weight (W) and age (A) or height (H), and there was a significant gender (G) difference in weight. Accordingly, in the following equation, we assumed a nonlinear function between weight on one left, and gender, age, and height on the right:

$$W=c\times A^{x}\times H^{y},$$

where c is a constant dependent on gender, and x and y are power factors of age and height, respectively. This nonlinear model structure is supported by well‐established allometric scaling theory, rigorous statistical theory, and successful applications in biomedical domains [40, 41, 55, 56, 57]. By performing a logarithmic transformation on both sides of the equation and taking gender as a dummy variable, we obtained the following log‐linear function:

lnW=a+x×lnA+y×lnH+b×G,

where a and b are constants and G is a dummy variable representing gender (for men G= 1, for women G= 0).

Based on the data of subgroup A, a, b, x, and y were calculated using stepwise multiple linear regression, and the OMAM equation of body weight was obtained:

lnW=2.7895+0.0526×lnA+2.1659×lnH+0.0326×G,

where the coefficient b≠0 indicates that gender has a significant effect on weight. After plugging the values of the dummy variable G into the above equation and performing exponential operations on both sides of the equation, we obtained the following weight equation for men:

(1) $$W=16.8121\times A^{0.0526}\times H^{2.1659},$$

and the weight equation for women:

(2) $$W=16.2729\times A^{0.0526}\times H^{2.1659}.$$

The significance tests of the model and coefficients were successfully passed with a goodness‐of‐fit adjusted $R^{2}$ of 0.6634, representing good fitting results and explanatory power. A full summary of the regression analysis and ANOVA results can be found in Tables S1 and S2.

The predicted value of body weight $W_{P}$ can be obtained after introducing age, height, and gender of individual subjects to the OMAM Equations (1) or (2). The corrected value $W_{C}$ can be obtained by dividing the measured value W by the predicted value $W_{P}$ [58]. Theoretically, if the hypothesis of the random error of linear regression is tenable, $\ln W_{C}=\ln W-\ln W_{p}$ should be close to a normal distribution with a mean of 0 and therefore the mean value of $W_{C}$ should be close to 1. In addition, as the random error is unpredictable, the corrected value $W_{C}$ should be linearly uncorrelated with the dependent variables of the regression equation including age, height, and gender. When the regression equation is set up and the coefficients a, b, x, and y are derived, the corrected value $W_{C}$ should be linearly correlated with the dependent variable W representing body weight. Based on the above analysis, we reckoned that the ideal equation for weight prediction should meet three conditions [40, 58, 59]: first, the mean value of $W_{C}$ is close to 1; second, the weight‐corrected value $W_{C}$ is correlated with the measured value W (*p *< 0.05); and third, $W_{C}$ is uncorrelated with age and height (*p *> 0.05), and no significant difference in $W_{C}$ is found between men and women (*p *> 0.05). Finally, we define the new criterion for determining overweight using the empirical 97.5th percentile of the corrected value $W_{C}$, provided that all the above conditions are satisfied.

### 6.3 Statistical Analysis

Measured values were expressed as median (IQR). Given the non‐normal distribution of some variables, Spearman rank correlation was used to assess the association between corrected values and measured weight, height, or age. Mann–Whitney *U* test was applied to examine gender differences in corrected values. The 95% confidence interval of the corrected value $W_{C}$ was estimated using the empirical percentile method based on its distribution in the population. The McNemar test was further used to compare the performance of the $W_{C}$‐ and BMI‐based criteria in identifying cardiometabolic diseases in the CAPITAL cohort. All statistical analyses were carried out by SPSS 23.0 software (SPSS, Inc., Chicago, IL) and *p* < 0.05 was regarded as statistically significant.

## Author Contributions

QZ, GHY, XYC, MZ, SW, CZ, XZ, and YZ developed the study protocol. XZ and YZ interpreted the data. QZ, XZ, and YZ drafted the manuscript and analyzed the data. All authors collected data and edited the manuscript. GHY, CZ, and MZ accessed and verified the underlying data. All authors had access to the data and accept responsibility for submitting the article for publication.

## Funding Information

This work was supported by grants of National Natural Science Foundation of China (82430015, 82527803), State Key R&D Program of China (2021YFF0501403), National Natural Science Foundation of Shandong (ZR2024ZD09), the Fundamental Research Funds for the Central Universities (202562003), the Taishan Scholars Program of Shandong Province (ts201511091), Qingdao Science and Technology Project (25‐1‐5‐smjk‐9‐nsh, 22‐3‐7‐smij‐6‐nsh), and Qingdao Key Clinical Specialty Elite Discipline (QDZDZK‐2022008).

## Ethics Statement

The EMINCA and CAPITAL studies were registered with the Chinese Clinical Trial Registry (ChiCTR‐OCS‐12002119 and ChiCTR‐TRC‐08000212, respectively), an authorized registry organization of the International Clinical Trial Registry Platform of the World Health Organization.

## Conflicts of Interest

The authors declare no conflicts of interest.

## Supporting information



Table S1: Summary of regression analysis.
Table S2: Model fitness and ANOVA results.

## Data Availability

Upon request, individual participant data that underlie the results reported in this article can be obtained by sending emails to the corresponding authors.
