# Supplementary material for: A New Mathematical Model to Index Body Weight in Healthy Chinese Han Adults
Source: MedComm (2020). 2026 Mar 2;7(3):e70649. doi: 10.1002/mco2.70649 (PMC12954139; doi:10.1002/mco2.70649)
Supplement: Supplementary file 1 — Table S1: Summary of regression analysis. Table S2: Model fitness and ANOVA results. [file MCO2-7-e70649-s001.docx]

**A new mathematical model to index body weight in healthy**

**Chinese Han adults**

Qing Zhang^1,2,3^, Gui-Hua Yao^2^, Xiang-Yun Chen^2^, Mei Zhang^1^, Xueying Zeng^4,5,*^, Shuping Wang^6,*^, Cheng Zhang^1,*^, Yun Zhang^1,*^

1. State Key Laboratory for Innovation and Transformation of Luobing Theory; The Key Laboratory of Cardiovascular Remodeling and Function Research, Chinese Ministry of Education, Chinese National Health Commission, Chinese Academy of Medical Sciences and Shandong Province; Department of Cardiology, Qilu Hospital of Shandong University, Jinan 250012, China
2. Department of Cardiology, Qilu Hospital (Qingdao), Cheeloo College of Medicine, Shandong University, Qingdao 266053, China
3. Department of Cardiology, Qilu Hospital, School of Medicine, Cheeloo College of Medicine, Shandong University, Jinan, Shandong, 250012, China
4. School of Mathematical Sciences, Ocean University of China, Qingdao, 266100, China
5. Laboratory of Marine Mathematics, Ocean University of China, Qingdao, 266100, China
6. Department of Endocrinology and Metabolism, Dongying People’s Hospital, Dongying 257091, China

****Correspondence***: Xueying Zeng (email: [zxying@ouc.edu.cn),](mailto:zxying@ouc.edu.cn),) Shuping Wang (email: [dqwg@163.com),](mailto:dqwg@163.com),) Cheng Zhang (email: [zhangc@sdu.edu.cn),](mailto:zhangc@sdu.edu.cn),) Yun Zhang (email: zhangyun@sdu.edu.cn)

Table S1: Summary of regression analysis

| Predictor | Coefficient (B) | SEs | $\text{t}$-value | $\text{P}$-value | VIF |
| --- | --- | --- | --- | --- | --- |
| LnHeight | 2.1659 | 0.0856 | 25.3153 | <0.001 | 2.1942 |
| LnAge | 0.0526 | 0.0080 | 6.6206 | <0.001 | 1.0996 |
| Gender | 0.0326 | 0.0082 | 3.9832 | <0.001 | 2.1053 |
| Constant | 2.7895 | 0.0570 | 48.9761 | <0.001 | - |

SEs: standard errors. VIF: variance inflation factors.

Table S2: Model fitness and ANOVA results

| Metric | Value |
| --- | --- |
| $\text{R}^{\text{2}}$ | 0.6646 |
| Adjusted $\text{R}^{\text{2}}$ | 0.6634 |
| F-statistic ($\text{F}$) | 563.3595 |
| Significance ($\text{P}$) | <0.001 |

**OMAM Calculation Instructions**

This section provides step-by-step instructions for calculating the corrected body weight (Wc) based on the OMAM model. The model adjusts body weight using age, height, and sex, and can be applied to clinical screening or epidemiological studies.

**1. OMAM Equation**

The OMAM equation takes the following form:

***W = c × A^x × H^y***

where:

- W is the observed body weight (in kg)

- A is the age (in years)

- H is the height (in m)

- c, x, and y are sex-specific coefficients estimated from the reference population

**2. Corrected Weight (Wc) Calculation**

The corrected weight Wc is calculated as the ratio of the observed weight (W) to the expected weight derived from the OMAM equation:

***Wc = W / (c × A^x × H^y)***

**3. Example Coefficients**

For example, in the healthy Chinese Han reference population:

- For males: c = 16.8121, x = 0.0526, y = 2.1659

- For females: c = 16.2729, x = 0.0526, y = 2.1659

**4. Sample Calculation**

Suppose a 50-year-old male has a height of 170 cm and a body weight of 72 kg. Then his Wc is calculated as follows:

Expected weight = 16.8121 × (50)^0.0526 × (1.7)^2.1659

Wc = 72 / Expected weight

If Wc > 1.1440, the individual is classified as overweight under the new OMAM-based criterion.

**5. Notes**

This calculation method can be implemented in spreadsheet software (e.g., Excel) or hospital information systems for automatic screening purposes. A user-friendly online calculator is available at: <https://xueyingzeng.github.io/OMAMcal/>
